# Risk factors and predictors of contralateral hip fracture after surgical treatment in elderly patients

**DOI:** 10.3389/fragi.2025.1633184

**Published:** 2025-08-18

**Authors:** Huan Yang, Yusong Yuan, Ruidong Ge, Lei Shi, Fangda Si, Ying Chen

**Affiliations:** Orthopedic Trauma, China-Japan Friendship Hospital, Beijing, China

**Keywords:** hip fracture, osteoporosis, bone mineral density, muscle imbalance, rehabilitation, geriatric trauma

## Abstract

**Background:**

Hip fractures are common in elderly patients, with some experiencing contralateral fractures. Even so, information on predictors of hip fractures in elderly adults is lacking. In this study, we investigated risk factors for contralateral hip fractures after surgical treatment of primary fractures.

**Methods:**

This was a prospective cohort study of 115 patients aged ≥65 years with low-energy hip fractures. The clinical parameters evaluated included age, sex, bone mineral density (BMD), T score, and hip flexor strength deficit. Patients were stratified into two groups: those with (n = 12) and those without contralateral fractures (n = 103).

**Results:**

Contralateral fractures occurred in 10.4% of the patients. Logistic regression revealed that age (OR = 1.08), reduced BMD (OR = 0.33), lower T score (OR = 1.45), and hip flexor imbalance (OR = 2.2) were significant predictors.

**Conclusion:**

A multimodal approach that integrates anti-osteoporosis therapy and targeted rehabilitation may reduce contralateral fracture risk in elderly patients.

## Introduction

The increasing prevalence of osteoporotic hip fractures in aging populations has drawn significant attention to contralateral hip fractures, which occur in 6.8%–16.0% of patients with osteoporotic hip fractures (Lau et al., 2014; Zidrou et al., 2023; Ryg et al., 2009). While previous research has focused predominantly on demographic and skeletal factors (Lau et al., 2014; Rathbun et al., 2017; Zhao et al., 2024), emerging evidence underscores the importance of biomechanical contributors, particularly postural instability and muscle weakness (Lau et al., 2014; Rathbun et al., 2017; Zhao et al., 2024; Gottschall and Kram, 2005; Akalan et al., 2016). The aim of this study was to combine biomechanical (hip flexor strength) and skeletal (BMD) parameters to identify multifactorial predictors of contralateral fractures. To our knowledge, this is the first prospective cohort study to integrate both biomechanical (hip flexor strength) and skeletal (BMD) parameters in the prediction of contralateral hip fractures.

### Study objectives and design

The investigation focused on systematically evaluating biomechanical, biochemical, and functional parameters, including 1) bone quality metrics (BMD and T scores via DXA); 2) neuromuscular functional capacity (hip flexor strength quantified through isokinetic dynamometry); 3) clinical outcomes (Harris Hip Scores); and 4) demographic/operative characteristics. A standardized data collection protocol was implemented across perioperative hospitalization and postdischarge follow-up (6-month intervals) to ensure longitudinal validity.

### Materials and methods

Consecutive patients who underwent surgical management for low-energy hip fractures at our Level I Trauma Center (January 2022-December 2023) and met the following criteria were included: 1. Age ≥65 years with low-energy trauma (fall from standing height); 2. Radiographically confirmed intertrochanteric (AO/OTA 31-A; n = 55) or femoral neck fractures (Garden II-IV; n = 60); and 3. Independent ambulation status prior to the fracture. Furthermore, patients were excluded if they met any of the following criteria: nonsurgical candidate, pathological fracture, neurological/musculoskeletal comorbidity, or visual impairment (best-corrected acuity <20/40). Postoperative Protocol: All patients received standardized anti-osteoporosis therapy and procedure-specific rehabilitation. In particular, in cases of PFNA fixation (n = 72), protected weight-bearing was implemented for ≥6 weeks, and in cases of arthroplasty (n = 43), immediate full weight-bearing post quadriceps-strength assessment (MMT grade IV) was implemented.

### Ethical compliance statement

This study was approved by the Ethics Committee of China-Japan Friendship Hospital (Approval No. 2023-KY-145), and written informed consent was obtained from all participants. All procedures involving human subjects were conducted in strict accordance with the ethical standards of the Declaration of Helsinki (2013 revision), the International Council for Harmonisation (ICH) Guidelines for Good Clinical Practice (GCP), and institutional review board (IRB) policies. Patient confidentiality and data anonymity were rigorously maintained throughout the study.

## Outcome assessment

### Operative protocol

Surgical procedures followed standard protocols for intertrochanteric fractures (PFNA fixation) and femoral neck fractures (hemiarthroplasty/THA).

### Postoperation protocol

All enrolled patients were routinely administered analgesics, anticoagulants and anti-osteoporosis medications according to standardized protocols. On postoperative day 2, targeted rehabilitation exercises focusing on quadriceps femoris activation, with particular emphasis on the vastus medialis oblique (VMO), were initiated. Patients were permitted to commence partial weight-bearing ambulation with full assistive support only after achieving grade IV muscle strength in the quadriceps femoris, as assessed by manual muscle testing (MMT).

### Follow-up

At the 6-month postoperative follow-up, comprehensive functional assessments were performed. Hip abductor function was evaluated using the standardized Trendelenburg test (Hardcastle and Nade, 1985), with positive findings (inability to maintain pelvic alignment during a single-leg stance), resulting in study exclusion. Hip flexor strength, represented by peak torque (PT), was measured using the BIODEX SYSTEM 4 PRO (Biodex Medical Systems, Inc., Shirley, NY) under (Gade et al., 2021) the supervision of licensed physical therapists. The testing protocol involved a unilateral stance with rapid contralateral leg elevation, measuring peak torque (ft-lb) and automatically calculating torque deficit percentages. Deficit severity was categorized as follows: 1) 1%–10%, within normal limits; 2) 11%–20%, indicating the need for rehabilitation; and 3) >20%, representing significant functional impairment. All measurements were recorded immediately postassessment to ensure data integrity and reliability.

### Study groups

The study population was stratified into two comparative cohorts on the basis of fracture characteristics: Group A (n = 12) consisted of patients presenting with contralateral hip fractures, and Group B (n = 103) comprised patients without contralateral involvement. A comprehensive comparative analysis was conducted between these cohorts. The variables in this analysis comprised multiple demographic and clinical parameters, including but not limited to age distribution, sex ratio, fracture classification according to the AO/OTA system, bone mineral density (BMD) in the hip region, T scores derived from dual-energy X-ray absorptiometry (DEXA) scans, length of hospital stay, Harris Hip Score (HHS) for functional assessment, and isokinetic measurements of hip flexor peak torque with corresponding deficit calculations.

### Statistical analysis

The sample size of 115 was determined on the basis of a power analysis (α = 0.05, β = 0.20) to detect a 15% difference between groups. All the statistical analyses were performed using PASW Statistics 21.0 software (IBM Corp., Armonk, NY, United States). Intergroup comparisons between Group A and Group B were conducted for multiple variables. Continuous variables, including age, time interval from injury to surgical intervention, length of hospital stay, bone mineral density (BMD), T score, Harris hip score (HHS), and isokinetic measurements of hip flexor peak torque (with associated deficit values), were analyzed using independent samples t tests following the confirmation of a normal distribution by means of Shapiro–Wilk tests. Categorical variables, such as sex distribution and fracture classification, were evaluated using Pearson’s chi-square (χ2) test or Fisher’s exact test, as appropriate. Missing data, which accounted for less than 5% of the total dataset, were excluded from the analysis under the assumption of being missing completely at random (MCAR). Variables demonstrating statistically significant differences (p < 0.05) in the univariate analysis were subsequently incorporated into multivariate logistic regression models to identify independent predictors. A two-tailed p value of <0.05 was considered statistically significant for all analyses.

## Results

Consecutive patients (N = 115) enrolled while 3 patients were excluded due to their request to withdraw consent. Contralateral fractures occurred in 10.4% of the patients (12/115) at a mean latency of 14.3 ± 3.8 months. Compared with the patients in Group B, those in Group A (contralateral fractures) were significantly older (84.3 vs. 79.1 years, *p* = 0.015), had a lower BMD (0.68 vs. 0.77 g/cm^2^, *p* = 0.021), and had higher proportion of cases of hip flexor deficit (22.5% vs. 14.6%, *p* = 0.032) (Table 1). Multivariate analysis revealed that age, T score, BMD, and hip flexor deficit were independent risk factors (Table 3).

**TABLE 1:** Univariate analysis of risk factors.

Parameter	Group A	Group B	Statistical significance
Age (years)	84.3 ± 5.1	79.1 ± 6.8	*t* = -2.16 *p* = 0.03
BMD (g/cm^2^)	0.68 ± 0.11	0.77 ± 0.09	*t* = 2.15 *p* = 0.03
T score	−3.1 ± 0.8	−2.0 ± 0.7	*t* = 4.94 *p* < 0.001
Peak torque (uninvolved)	58.7 ± 7.9	63.2 ± 8.1	*t* = 2.15 *p* = 0.03
Hip flexor deficit (%)	22.5 ± 5.3	14.6 ± 4.1	*t* = −2.01 *p* = 0.047

BMD: bone mineral density; PFNA: proximal femoral nail antirotation; MMT: manual muscle testing.

### Postoperative outcomes and fracture epidemiology

The mean hospitalization duration was 8.7 ± 3.2 days (range: 3–22) in the prospective cohort, and the injury-to-surgery interval was 1.8 ± 0.9 days (range: 1–3), with no intergroup differences (*p* > 0.05). Systematic follow-up over 6–20 months revealed a contralateral fracture incidence of 10.4% (12/115), indicating a balanced sex ratio, comprising 6 males and 6 females, representing an equal proportion (50%) in Group A. Fracture patterns were comparable between intertrochanteric (10.9%, 6/55) and femoral neck fractures (10.0%, 6/60). Contralateral hip fractures occurred at a mean latency of 14.3 ± 3.8 months postoperatively (range: 9–20).

### Surgical and rehabilitation protocols

All patients received standardized anti-osteoporosis therapy (calcium/alfacalcidol) and procedure-specific rehabilitation:

• PFNA fixation (n = 72): Protected weight-bearing for ≥6 weeks with progressive quadriceps strengthening.
• Arthroplasty (n = 43): Immediate full weight-bearing upon achieving MMT grade IV quadriceps strength. Postoperative hip mobility was restricted to −10°–90° during the first month, supplemented by supervised isometric exercises initiated within 24 h postoperatively.

### Comparative analysis of risk factors

Univariate analysis revealed significant disparities between the contralateral fracture (Group A, n = 12) and control (Group B, n = 103) cohorts (Table 1).

No significant differences were observed between Group A and Group B in terms of sex, length of hospital stay, fracture type, Harris score, or involved hip flexor peak torque (Table 2).

**TABLE 2:** Comparison of clinical characteristics between group A and group **B**.

Variable	Group A (n = 12)	Group B (n = 103)	Statistical value	*p* value
Sex (Male/Female)	4 (33.3%)/8(66.7%)	29 (28.2%)/74(71.8%)	χ^2^ = 3.45	0.06
Length of Hospital Stay (days)	9.8 ± 4.2	8.5 ± 3.1	*t* = −0.78	0.44
Fracture Type				
- Intertrochanteric Fracture	5 (41.7%)	50 (48.5%)	χ^2^ = 6.356	0.70
- Femoral Neck Fracture	7 (58.3%)	53 (51.5%)		
Harris Score	81.0 ± 10.88	79.95 ± 10.73	*t* = −0.54 0.60	
Peak Torque (involved)	55.1 ± 7.2	52.8 ± 6.7	*t* = −1.23 0.22	

The logistic regression analysis revealed that age, lower T scores, reduced BMD, and greater hip flexor deficit were significant risk factors for contralateral hip fractures. These findings highlight the importance of addressing both skeletal and biomechanical factors in the prevention and management of contralateral hip fractures in elderly patients (Table 3). Although univariate analysis suggested a trend toward higher contralateral fracture rates in males (33.3% vs. 28.2%), this association did not reach statistical significance in multivariate modeling (p = 0.603).

**TABLE 3:** Multivariate logistic regression analysis of independent risk factors for contralateral hip fracture**s**.

Variable	β	SE	*p* value	OR	CI
Age	0.072	0.29	**0.012**	**1.08**	[1.02,1.15]
Sex	0.210	0.40	0.603	1.23	[0.56, 2.71]
Hospital Stays	−0.015	0.038	0.692	0.98	[0.91,1.06]
Fracture type	0.102	0.189	0.589	1.11	[0.76,1.61]
BMD	−1.139	0.518	**0.028**	**0.32**	[0.11,0.89]
T score	0.372	0.140	**0.008**	**1.45**	[1.10,1.92]
Harris score	−0.008	0.014	0.567	0.99	[0.97,1.02]
PT (Involved)	−0.015	0.02	0.454	0.98	[0.94,1.03]
PT (Uninvolved)	−0.021	0.018	0.244	0.99	[0.94,1.02]
PT deficit (%)	0.788	0.286	**0.006**	**2.2**	[1.25,3.86]

BMD: bone mineral density; PT: peak torque; PT: deficit: Peak torque deficit; *Only variables with p < 0.05 were considered statistically significant risk factors in multivariate analysis*.

**FIGURE 1:**
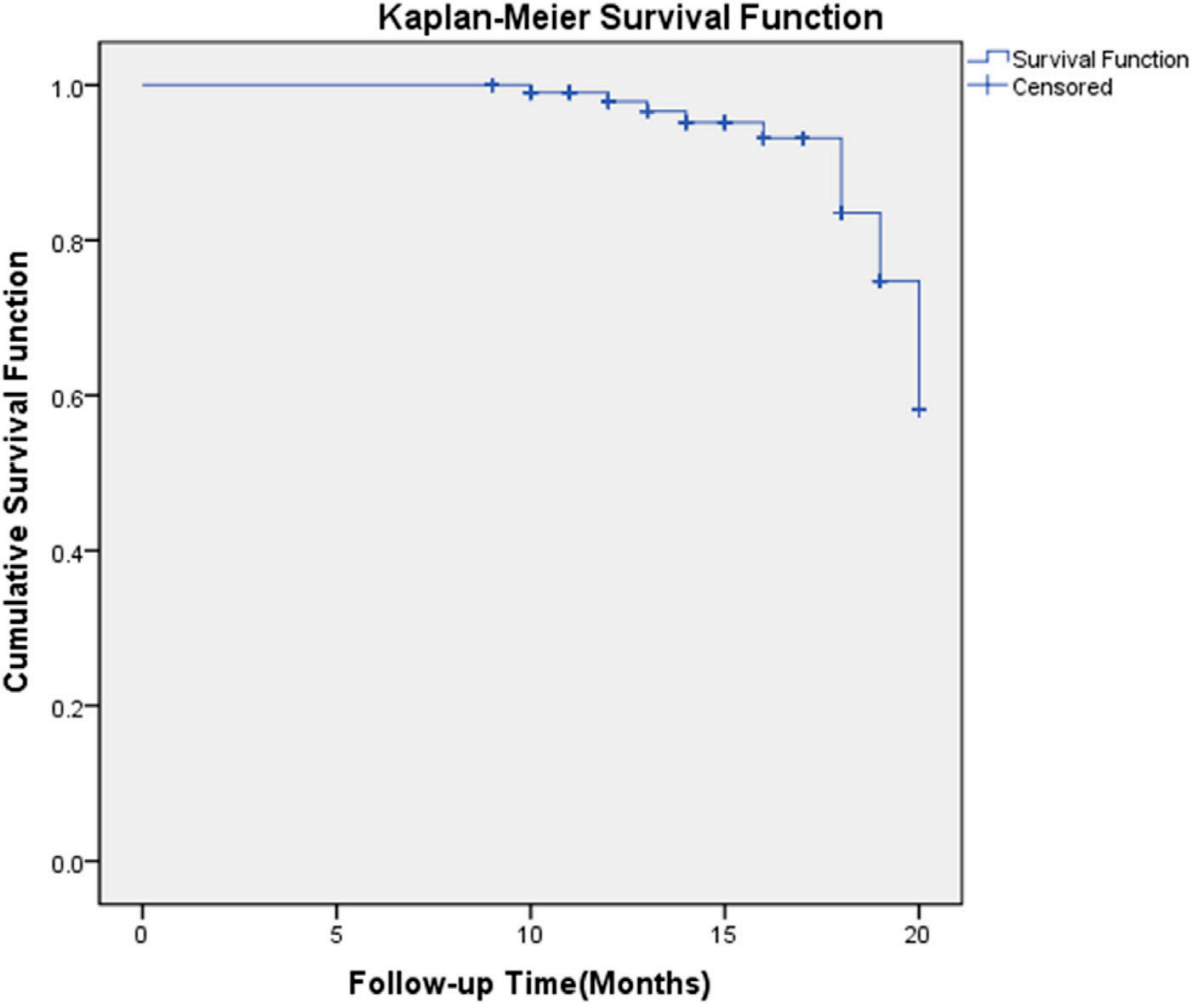
Kaplan-meier survival curve for contralateral hip fracture incidence.

Kaplan-Meier analysis revealed a cumulative contralateral fracture incidence of 10.4% at 20 months (Figure 1).

The Kaplan-Meier curve illustrates the cumulative incidence of contralateral hip fractures over a 20-month follow-up period in elderly patients following surgical treatment of primary hip fractures. The x-axis represents time in months, and the y-axis shows the cumulative survival probability (i.e., the proportion of patients without contralateral fractures). The curve demonstrates a gradual decline in survival probability, with a cumulative incidence of 10.4% at 20 months. Censored data points (indicated by “+” symbols) represent patients who experienced a contralateral hip fracture. This analysis highlights the temporal pattern of contralateral fracture risk, emphasizing the need for extended monitoring and secondary prevention strategies beyond the first-year post-surgery.

### Factors associated with contralateral hip fractures

Bone mineral density (BMD) measurements revealed a mean of 0.68 ± 0.11 in Group A and 0.77 ± 0.09 in Group B (*t* = 2.15*, p* = 0.03), whereas T scores of −3.1 ± 0.8 in Group A and −2.0 ± 0.7 in Group B indicated more severe osteoporosis in patients with contralateral fractures (*t* = 4.94, *p* < 0.001). The musculoskeletal assessment revealed that the percent deficit in hip flexor strength was significantly greater in Group A (22.5 ± 5.3) than in Group B (14.6 ± 4.1); *t* = −2.01, *p* = 0.047), suggesting greater bilateral muscle imbalance in patients with contralateral fractures.

## Discussion

The synergistic effect of advanced age and reduced BMD underscores the critical role of osteoporosis management in contralateral fracture prevention (Leslie and Morin, 2014; Gregg et al., 2000; Ryg et al., 2009), as evidenced by our findings (Group A: BMD 0.68 vs. Group B: 0.77 g/cm^2^, p = 0.03). A lower T score (−3.1 vs. −2.0, *p* < 0.001) aligns with prior evidence that age-related bone loss exacerbates fracture risk (Fujita et al., 2022). The mean latency period of 14.3 months postoperatively highlights a critical window for secondary prevention, potentially linked to delayed bone remodeling and altered weight-bearing mechanics (Ramisetty et al., 2015; Rathbun et al., 2017). Extended follow-up protocols beyond 12 months may improve monitoring efficacy. Notably, hip flexor strength imbalance (OR = 2.2) emerged as a stronger biomechanical predictor, suggesting targeted rehabilitation to mitigate gait instability and fall risk (Fujita et al., 2022; Gade et al., 2021; Akalan et al., 2016).

The findings of this study provide critical insights into the multifactorial nature of contralateral hip fractures in elderly patients, highlighting several key areas for clinical consideration and future research.

### Age/BMD interaction

The significant correlation between advanced age and reduced bone mineral density (BMD) underscores the synergistic effect of these factors in increasing contralateral hip fracture risk (Leslie and Morin, 2014; Gregg et al., 2000; Ryg et al., 2009; Zhao et al., 2024; Vochteloo et al., 2012; Fujita et al., 2022). Our data show that in patients with contralateral fractures, compared with those of the controls, the BMD values of the patients are significantly lower (0.68 ± 0.11 g/cm^2^), and the mean age of the patients is significantly higher (84.3 ± 5.1 years). This finding aligns with those reported in the literature indicating that age-related bone loss exacerbates fracture susceptibility, particularly in the proximal femur (Leslie and Morin, 2014; Gregg et al., 2000; Ryg et al., 2009; Fujita et al., 2022). The observed T score disparity (−3.1 ± 0.8 vs. −2.0 ± 0.7, *p* < 0.001) further emphasizes the need for aggressive osteoporosis management in this population.

### Temporal pattern of contralateral fractures

The mean latency period of 14.3 ± 3.8 months for contralateral fractures suggests a critical window for secondary prevention (Zhu et al., 2014). This temporal pattern may reflect the combined effects of postsurgical bone remodeling, altered weight-bearing mechanics, and potential delays in achieving optimal BMD through pharmacological intervention (Zhao et al., 2024; Vochteloo et al., 2012). The clustering of contralateral hip fractures between 9 and 20 months postoperatively warrants the consideration of extended monitoring protocols beyond the conventional 12-month follow-up period (Ryg et al., 2009; Zhao et al., 2024; Vochteloo et al., 2012; Fujita et al., 2022).

### Biomechanical factors

The significantly greater hip flexor peak torque deficit in the contralateral fracture group (22.5% ± 5.3% vs. 14.6% ± 4.1%, *p* = 0.047) highlights the biomechanical consequences of muscular imbalance. This deficit may contribute to altered gait patterns and increased fall risk, creating a vicious cycle of instability and fracture susceptibility (OR = 2.2) (Zhao et al., 2024; Fujita et al., 2022; Zhu et al., 2014). The integration of targeted hip flexor strengthening into rehabilitation protocols may mitigate these risks (Fujita et al., 2022; Methenitis et al., 2016).

### Protocol-specific rehabilitation outcomes

Our protocol-specific approach, which differentiates between PFNA fixation and arthroplasty patients, demonstrated the importance of tailored rehabilitation strategies (Kahn et al., 2013; Zhao et al., 2024; Vochteloo et al., 2012; Fujita et al., 2022; Parker and Handoll, 2010; Bhandari et al., 2003). The immediate weight-bearing protocol for arthroplasty patients, contingent upon achieving MMT grade IV quadriceps strength, appeared to facilitate earlier functional recovery without compromising surgical outcomes (Fujita et al., 2022; Bhandari et al., 2003). Conversely, the protected weight-bearing regimen for PFNA patients likely contributed to the observed fracture consolidation rates (Zhao et al., 2024; Parker and Handoll, 2010; Bhandari et al., 2003; Zhu et al., 2014).

### Multivariate regression analysis of risk factors

Preliminary multivariate analysis revealed three independent predictors of contralateral fracture risk (Zhao et al., 2024; Zhu et al., 2014): age ≥80 years (OR = 1.08, 95% CI: 1.02, 1.15), BMD≤0.5 (OR = 0.32, 95% CI: 0.11, 0.89), T score ≤ −2.5 (OR = 1.45, 95% CI: 1.10–1.92), and hip flexor deficit ≥20% (OR = 2.2, 95% CI: 1.25–3.86). These findings underscore the multifactorial nature of contralateral hip fracture risk and the need for comprehensive risk assessment tools (Leslie and Morin, 2014; Zidrou et al., 2023; Ryg et al., 2009; Zhao et al., 2024; Fujita et al., 2022; Kanis et al., 2008; Compston et al., 2017).

### Longitudinal analysis of functional recovery

The Harris Hip Score (HHS), while widely used, has notable limitations, including subjectivity in patient-reported outcomes, insensitivity to subtle functional changes (Ramisetty et al., 2015; Wamper et al., 2010), and inadequate assessment of physical activity levels, particularly muscle strength (Wamper et al., 2010). These limitations underscore the need for complementary assessment tools in high-risk populations. The lack of significant HHS differences between groups (Table 2) likely reflects its limited sensitivity for detecting subtle functional deficits, as previously documented (Ramisetty et al., 2015; Wamper et al., 2010). Consequently, researchers and clinicians should consider alternative or supplementary scoring systems when evaluating hip-related outcomes.

### Subgroup analysis based on fracture classification

The comparable contralateral fracture rates between intertrochanteric (10.9%) and femoral neck fractures (10.0%) suggest that fracture morphology may be less predictive of contralateral fracture risk than systemic factors such as BMD and muscular function. However, the small subgroup sizes and single-center study design limit definitive conclusions, warranting larger-scale investigations (Zhao et al., 2024; Vochteloo et al., 2012). Potential measurement errors in hip flexor strength assessment and a follow-up period limited to 20 months, which may limit generalizability.

## Clinical implications

These findings not only advance risk stratification for contralateral hip fractures but also highlight broader implications for geriatric musculoskeletal health. The interplay between muscle imbalance and bone fragility may extend to other fragility fractures (e.g., vertebral or distal radius fractures), suggesting a unified framework for sarcopenia-osteoporosis management in aging populations. Lack of appropriate physical therapy after the first fracture is a significant risk factor for shorter intervals between first and second hip fractures in geriatric patients (Saglam et al., 2024). Furthermore, integrating biomechanical biomarkers (Antico et al., 2012) (e.g., muscle deficit quantification) with systemic inflammatory profiles could catalyze interdisciplinary collaborations spanning orthopedics, geriatrics, and rehabilitation medicine. To bridge current evidence gaps (Koot et al., 1996; Salo and Logomarsino, 2011; Liu, 2014; Methenitis et al., 2016), future research should prioritize three avenues: 1) cost-effectiveness analyses of population-level osteoporosis screening informed by fracture biomechanical risk, 2) development of AI-driven predictive models that synthesize radiological, biochemical, and functional mobility data, and 3) clinical trials evaluating whether neuromuscular re-education protocols—successful in stroke rehabilitation—can be adapted to mitigate post-fracture imbalance. Such translational efforts may redefine preventive care paradigms beyond traditional bone-centric approaches.

## Data Availability

The datasets generated and/or analyzed during the current study are available in the figshare repository, https://doi.org/10.6084/m9.figshare.28620911. Further inquiries can be directed to the corresponding author.
